# Out-of-season spawning of largemouth bass in a controllable recirculating system

**DOI:** 10.3389/fphys.2023.1175075

**Published:** 2023-04-24

**Authors:** Chen-Hao Hu, Han-Qing Bie, Zi-Yi Lu, Yang Ding, He-He Guan, Long-Hui Geng, Shuai Ma, Yuan-Xiang Hu, Qi-Xue Fan, Zhi-Gang Shen

**Affiliations:** ^1^ College of Fisheries, Key Lab of Freshwater Animal Breeding, Ministry of Agriculture and Rural Affairs/Key Lab of Agricultural Animal Genetics, Breeding and Reproduction of Ministry of Education/Engineering Technology Research Center for Fish Breeding and Culture in Hubei Province/Engineering Research Center of Green Development for Conventional Aquatic Biological Industry in the Yangtze River Economic Belt of Ministry of Education, Huazhong Agricultural University, Wuhan, China; ^2^ HuBei HuangYouYuan Fishery Development Limited Company, Wuhan, China

**Keywords:** largemouth bass, out-of-season spawning, ovary development, energy allocation, recirculating system

## Abstract

Largemouth bass (LMB) production exceeded 0.7 million tons in 2021 and has become one of the most important freshwater aquaculture species in China. The stable and fixed culture cycle led to regular and drastic price fluctuation during the past decade. Strong price fluctuation provides opportunities and challenges for the LMB industry, and out-of-season spawning (OSS) and culture will provide technical support for the opportunities. To induce OSS at a low cost, we established a controllable recirculating system that allows precise thermo-photoperiod manipulation. In the system, four experimental groups were assigned, 18NP (18°C overwintering water temperature, natural photoperiod), 18CP (18°C overwintering water temperature, controlled photoperiod), 16CP (16°C overwintering water temperature, controlled photoperiod), and NTNP (natural water temperature and natural photoperiod), to determine the effects of chilling temperature and photoperiod on spawning performance. OSS was observed in all the experimental groups without significant differences, except NTNP. The manipulated broodstock can re-spawn 3 months later in the next spring in advance. Further analysis of the volume percentage of different stages of oocytes provides a base for excellent regression between the volume percentage of the primary growth stage, cortical alveoli stage, vitellogenesis/maturation stage, and gonadal development/maturation. The results suggest that the volume percentage of oocytes is a better indicator of gonadal development and maturation than the gonadosomatic index. We also found that LMB prefers palm fiber as a spawning nest over gravel. The findings of this work provide important technique guidance for practical OSS of the LMB aquaculture industry and standardization of ovary development and maturation in fish with asynchronous developmental oocytes.

## 1 Introduction

**FIGURE 1:**
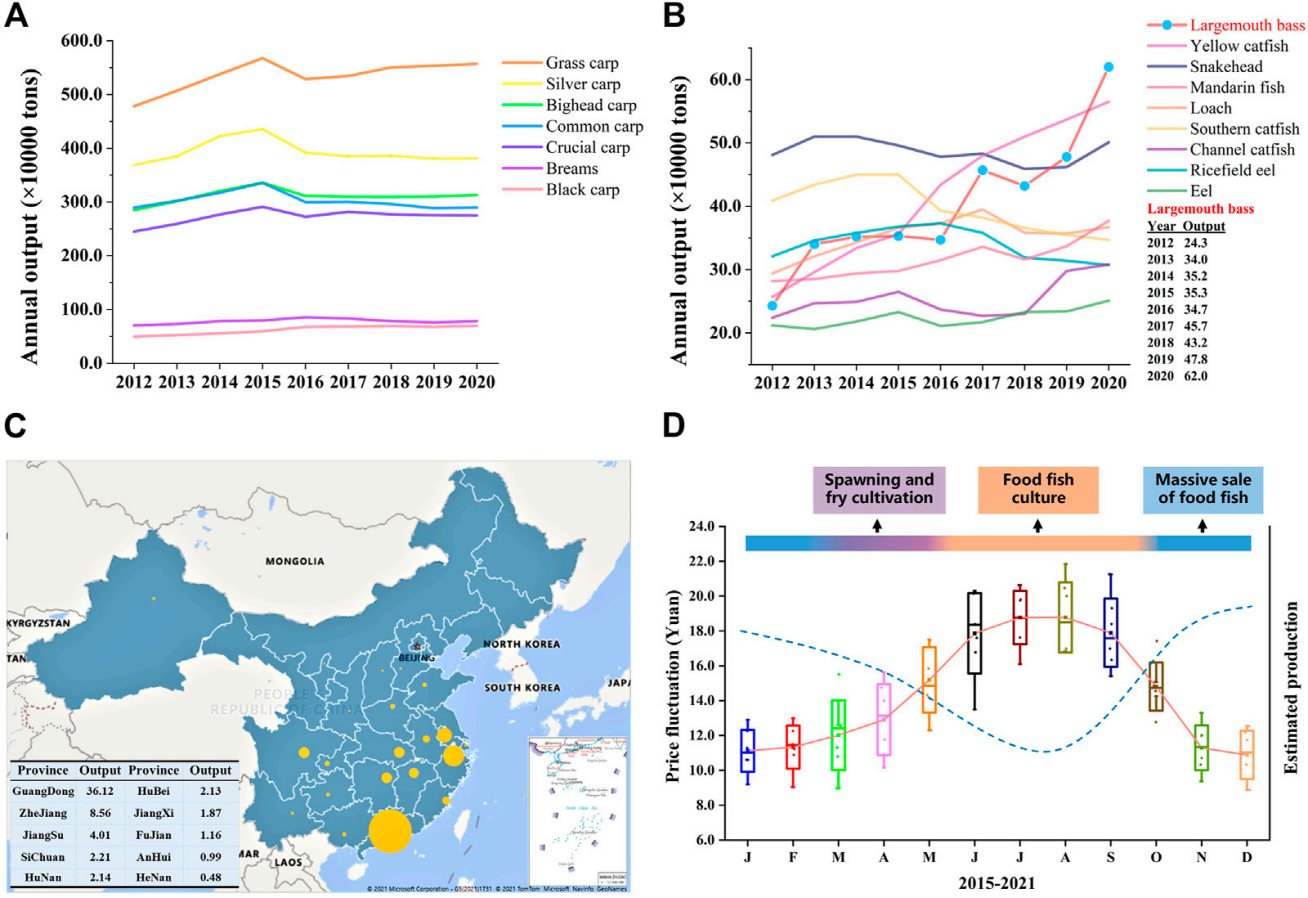
Annual production change of the top 16 freshwater fish species and production distribution and price fluctuation of largemouth bass in China. **(A, B)** The annual production output of the top 16 fish species (>0.2 million tons), including seven staple/conventional freshwater fish species and nine special freshwater species, from 2012 to 2020 in China. Tilapias are not listed because they are mainly exported. (**C)** Aquaculture production of largemouth bass in different provinces of China in 2020. The size of the bubble refers to the amount of production for the corresponding province. The top 10 producers of largemouth bass are listed in the table. Annual production data were obtained from the China Fishery Statistical Yearbook (China Bureau of Fisheries). (**D)** Annual average price fluctuation of largemouth bass and estimated production (dotted line), showing the supply–demand relationship. Each box represents the price data of the same month from 2015 to 2021, and the solid line represents the average annual price fluctuation trend. Data for the price, representing pond-side price or purchasing price which is the price that the dealer offers to the fish farmer, were collected from the China Fisheries Channel website (http://www.fishfirst.cn/) and China Aquaculture website (http://www.shuichan.cc/).

Aquaculture, as the main source of blue food and the fastest-growing food-producing sector in the world, plays important roles in poverty reduction and promoting rural revitalization, food security, and good nutrition, reducing carbon emissions, and combatting ecosystem overexploitation worldwide (Naylor et al., 2000; Tidwell and Allan, 2001; Gui et al., 2018; Han et al., 2018; Editorial, 2021; Golden et al., 2021). China has been a major fish producer since the 1990s and accounted for 35% of the global fish production in 2018 (FAO, 2020). Freshwater aquaculture makes up 59% of the total aquaculture production in 2020 and occupies the main seafood consumption in China. Staple/conventional freshwater fish species (SFS, grass carp, silver carp, bighead carp, black carp, common carp, crucian carp, and bream), which are born with intermuscular bone, account for about two-thirds of the total freshwater production during the past 2 decades in China’s freshwater aquaculture industry (China Fishery Statistical Yearbook; Fishery Bureau, Ministry of Agriculture, P. R. China). With the development of society and the improvement of people’s living standards, the production and consumption demand for SFS has decreased since 2016 (Figure 1A), and the consumer preference has switched to fish species with none/less intermuscular bone and better flesh quality (Figure 1B) (Newton et al., 2021). More importantly, farming high-value fish, e.g., largemouth bass, yellow catfish, and mandarin fish (Figure 1B), means more economic income for fish farmers (Hussein et al., 2020; Yu et al., 2021; 2022).

The largemouth bass (LMB), *Micropterus salmoides*, has been the most popular and widely distributed game fish since 1975 in the United States of America (Wallus and Simon, 2008; Cooke and Philipp, 2009). It has become one of the most important aquaculture species in China because of the aquaculture (e.g., rapid growth, short culture cycle, and strong adaptability) and dietary advantages (excellent flesh quality and no intermuscular bone) (Bai and Li, 2018; 2019; Shen et al., 2018; Hussein et al., 2020). As the Chinese saying goes, no fish, no feast. LMB has become the main choice for fish diets in many kinds of dinners and banquets in Central, South, and East China in the past years and the main raw material for pre-made food in recent years. After the success of large-scale induced spawning, pond aquaculture, and formulation of expanded feed, LMB has been farmed in 27 provinces, and the aquaculture production increased from 0.243 million tons in 2012 to 0.702 million tons in 2021 with an average growth rate of 19.4% (Figures 1B, C). As the first point of introduction of LMB to the Chinese mainland in the 1980s, Guangdong has remained the major LMB producer and yields more than 55% percent of global LMB production. Guangdong plays the role of a pilot and provides important support to the LMB aquaculture development of other provinces. Centralized production and unitary consumption size in the market (400–600 g) result in a stable and fixed culture cycle and generate an annually stable supply–demand relationship, consequently leading to regular and drastic price fluctuation during the past years (Figure 1D). For example, March and May are the main periods for induced spawning and fry cultivation, and June to October is the main period for food fish culture. Therefore, food fish is sold massively from November to February which leads to comparatively low market prices (pond price) (Hussein et al., 2020).

Strong price fluctuation offers a great opportunity for the out-of-season culture of LMB, which means higher economic benefits and will help relieve the imbalanced supply–demand relationship. For example, the price of newly hatched LMB larvae in November and December is ten times higher than that produced in March and April, and the price of food fish in the summer is approximately twice the price in the winter of 2021 in China (Figure 1D). Out-of-season spawning (OSS), the most important foundation for out-of-season culture, can be realized by three technical approaches. First, OSS is realized through the compression of an annual spawning cycle and the redevelopment of gonads, which is named the compression method. Second, OSS can be achieved by the postponement of spawning activity by maintaining a water temperature lower than the spawning temperature, which is named the postponement method. Lastly, the OSS is realized through the induced spawning of offspring produced by OSS, which is named the new-fish method. The compression method is the most commonly used approach and succeeds in several groups of fish, including salmonids, percids, moronids, gadids, and cyprinids (Malison et al., 1998; Migaud et al., 2002; Wang et al., 2010; Żarski et al., 2019).

The success of OSS mainly relies on the proper manipulation of temperature and photoperiod (Wang et al., 2010), which is resource-dependent or resource-consuming. For example, OSS of LMB has been reported, by several research groups (Brauhn et al., 1972; Carlson, 1973; Jackson, 1979; Matthews and Stout, 2013), to produce large-size fingerlings for huge recreational fisheries in the United States of America. We have previously conducted OSS of LMB using deep water reservoir resources and successfully produced LMB fry in September and October (Hussein et al., 2020). However, the large-scale OSS of LMB in our previous work and in the industry is costly and uncontrollable, as with other approaches that are dependent on low water temperature either from a deep well or reservoir. These OSS methods require high consumption of electric power, expensive facilities, and/or limited resource (e.g., cold water or warm water). Therefore, a controllable and cost-effective protocol for OSS in LMB is urgently needed.

In the present work, we designed a cost-effective and controllable recirculating system and investigated the effects of temperature and photoperiod manipulation on the OSS of LMB. We successfully realized the OSS of LMB in a small controllable system.

## 2 Methods and materials

This study and all experimental procedures involving animals were performed according to the protocol approved by the Animal Care and Use Committee (ACUC) of Huazhong Agricultural University. The ACUC requires that experimental animals need to be treated kindly, anesthetized before sampling, and the sampling number should be minimized. Individuals were anesthetized before sampling using 7–10 mg L^−1^ eugenol. The sampling number was fully designed before the experiment.

### 2.1 Parents’ source

LMB broodstock was obtained from HuBei HuangYouYuan Fishery Development Limited Company (located in LuHu, Wuhan). These fish were the selected offspring of the first accredited LMB new variety “Youlu No.1” (by the National Committee for Examination and Approval of Primary and Improved Aquatic Products).

### 2.2 Design of a controllable and low-cost recirculating system

**FIGURE 2:**
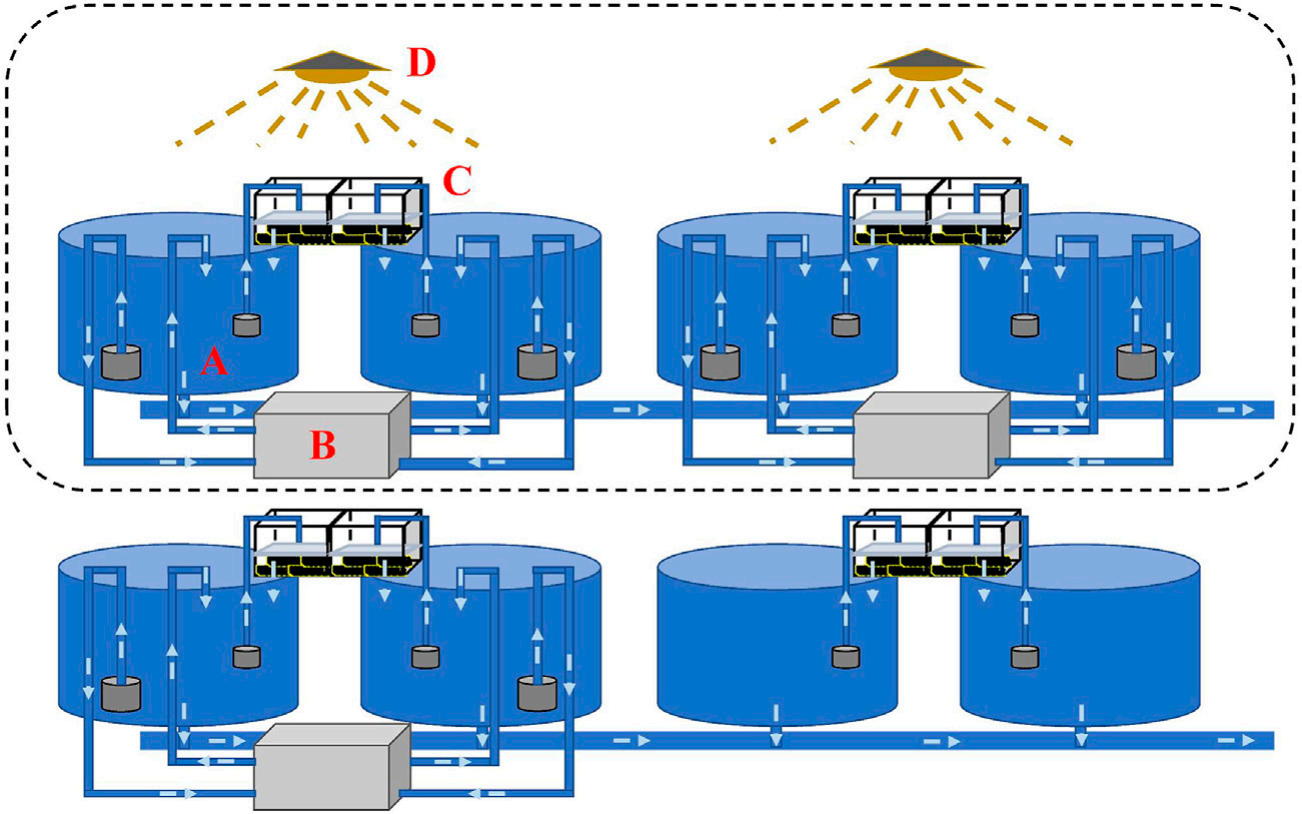
Design of a controllable recirculating aquaculture system. Each recirculating system/tank consists of a culturing tank [**(A)** diameter = 0.9 m], a refrigerating machine **(B)**, a filtration system **(C)**, and a timing light **(D)**. Water temperature and photoperiod can be manipulated using the refrigerating machine and the timing light, respectively. The water volume in each recirculating tank was 1,100 L.

To realize OSS in the autumn, water temperature needs to be decreased to simulate wintering during the summertime. Decreasing the temperature in an earthen or concrete pond to simulate wintering requires high-power facilities or a large number of groundwater resources, which is costly and environmentally unfriendly. Therefore, we build a small controllable recirculating system incorporating a culturing tank, refrigerating machine, filtration system, and timing light (Figure 2). The system allows high density during the wintering period and low density making the best use of warm weather during the pre-spawning and spawning periods, to largely reduce the power for temperature control. Water temperature and photoperiod were manipulated through the pump-in and pump-out of water using the refrigerating machine and the 24-hour timer connected to an LED light, respectively. The water volume of each independent recirculating system including a cone-bottom round culturing tank (diameter = 0.9 m), a refrigerating machine, and a filtration system was 1,100 L. A black insulation film was set up in a cuboid shape to cover experimental tanks for water temperature and photoperiod manipulation. To maintain good water quality, the filter cotton was replaced every 3 days, and culturing tanks were siphoned every day.

### 2.3 Out-of-season and in-season spawning

#### 2.3.1 Out-of-season spawning

The methodology of OSS is realized by compressing the annual reproductive cycle (especially the changes of thermo-photoperiod) into several months to simulate and promote gonadal development and spawning during the non-reproductive season, referring to the LMB annual reproductive cycle under natural conditions. Regimes for thermo-photoperiod manipulation refer to our previous work in Centrarchidae species (Shen et al., 2016; 2018; Hussein et al., 2020; Cui et al., 2021) with adjustment. LMB broodstock of 1+ years old, which had spawned during the reproductive season in April and May, was harvested from a pond and transferred to the indoor recirculating system. Two hundred mature LMBs, with an average weight of 399.8 G, were randomly distributed evenly in eight tanks (25 ind. per tank). Four groups, i.e., **18NP** (18°C overwintering water temperature, natural photoperiod), **18CP** (18°C overwintering water temperature, controlled photoperiod), **16CP** (16°C overwintering water temperature, controlled photoperiod), and **NTNP** (natural water temperature and natural photoperiod), were arranged with two replicates for each. The average initial weights of **18NP**, **18CP**, **16CP**, and **NTNP** groups were 400.4 ± 69.6, 399.0 ± 76.6, 399.8 ± 76.8, and 389.9 ± 58.2 g, respectively.

**FIGURE 3:**
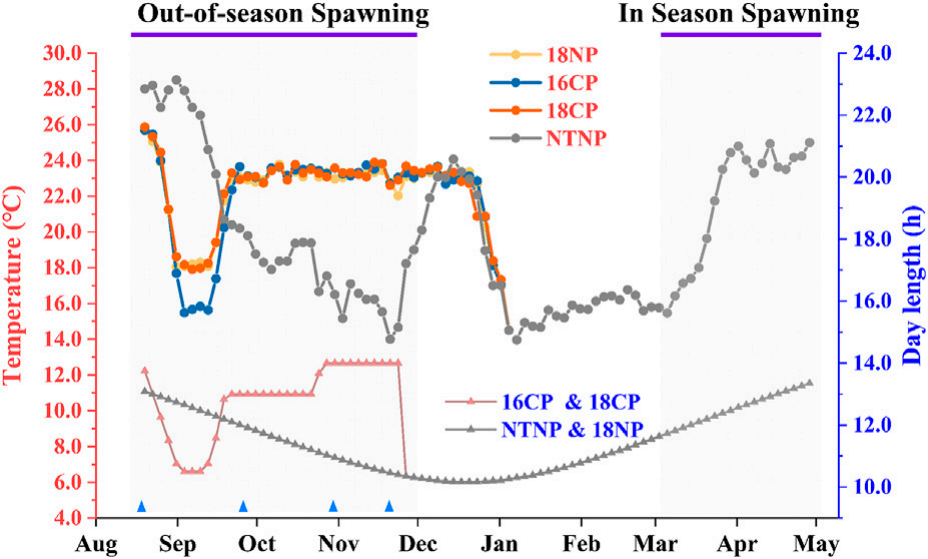
Thermo-photoperiod manipulation in out-of-season and in-season spawning of largemouth bass. The experiment was divided into four groups, i.e., **18NP** (18°C overwintering water temperature, natural photoperiod), **18CP** (18°C overwintering water temperature, controlled photoperiod), **16CP** (16°C overwintering water temperature, controlled photoperiod), and **NTNP** (natural water temperature and natural photoperiod). The average water temperature and photoperiod every 3 days are indicated by the upper and lower lines, respectively. Blue triangles indicate the four sample points at Stage 1, Stage 2, Stage 3, and Stage 4, which are described in the *2.4 Sampling and histological and energy analysis* section.

The broodstocks were transferred from the in-pond raceway system into the in-door system on 16 July and fed with special expanded formula feed enriched with multivitamins for 1 month to ensure normal development and maturation of gonads. Water temperature and photoperiod manipulation are shown in Figure 3. Briefly, the water temperature was gradually reduced at a rate of 1°C per day to 18°C or 16°C for the 18NP and 18CP or 16CP groups, respectively. To reduce the cost of water temperature control, the targeted low overwintering water temperatures were maintained for 2 weeks and then increased at a rate of 1°C per day to the optimal local spawning water temperature (23°C). At the same time, the photoperiod for 18CP and 16CP groups was adjusted as the similar regimen as water temperature, i.e., reduced, maintained, and increased, which refers to the local annual reproductive photoperiod cycle. The water temperature and photoperiod in the NTNP group were maintained naturally until the spawning period of other groups. Water temperature was gradually increased in the NTNP group to stimulate spawning. The photoperiod in the 18NP group was maintained naturally. The range of temperature in the NTNP group was from 12.0°C to 28.8°C. We measured the light intensity from the edge to the center of the water surface, and it was from 13 Lux to 338 Lux in the natural condition and from 5 Lux to 312 Lux for the controllable system.

The broodstock in each group was distributed into three tanks (0.9 m diameter, 1,000 L, 10 ind. per tank) when the water temperature reached 23°C. Five males and five females were randomly selected (distinguished by squeezed sperm from cloacal opening) and mated in each spawning tank. Two types of spawning nests, palm fiber sheet (PFN, 0.030 m × 0.033 m, ∼0.1 m^2^ dimension) and round basin with gravel (GN, diameter 0.36 m, ∼0.1 m^2^ dimension), were placed in spawning tanks to investigate the preference for the spawning nest of LMB when the water temperature reached 23°C. Two palm fiber nests and two gravel nests crisscross in each spawning tank. At this time, the broodstock was unceasingly fed with LMB special expanded formula feed enriched with multivitamins for 1 month to ensure good maturation of the gonads. Spawning agent LHRH-A2 was injected thrice with a dose of 3 μg kg^-1^ every 6 days starting from 26 October. Spawning nests were checked twice a day at 7–8 a.m. and 8–9 p.m. Spawning nests with eggs were photographed and checked for egg amount and fertilization rate.

#### 2.3.2 In-season spawning

Water temperature and photoperiod gradually recovered to natural conditions after the termination of OSS in December. Therefore, the spawned broodstock underwent overwintering once again. In the March of next year of OSS, broodstocks in the three experimental groups (18NP, 18CP, and 16CP) were redistributed into two spawning tanks with five males and five females in each. Water temperature was increased to 23°C using heaters with a rate of 0.5°C per day. Photoperiod was maintained naturally. Two palm fiber nests and two gravel nests crisscross in each spawning tank when the water temperature reached 23°C. At this time, spawning agents LHRH-A2 and DOM were injected once with a dose of 3 μg kg^-1^ and 5 mg kg^-1^, respectively. Spawning nests were checked twice a day at 7–8 a.m. and 8–9 p.m.

### 2.4 Nutrient enrichment of broodstock and management

To maintain good water quality and prevent a pathogenic outbreak in the small recirculating system, the broodstock was fed with the LMB special expanded formula feed in the whole course of rearing. The LMB broodstock was enriched with vitamin A (3500 IU kg^-1^), vitamin C (2 g kg^-1^), and vitamin E (60 mg kg^-1^) to ensure normal development and maturation of gonads. Vitamin C was dissolved in 20 mL pure water and mixed with 10 mL 95% ethanol which contains vitamins A and E. The 30-mL suspension liquid was then evenly sprayed on the surface of 1 kg feed. The enriched feed was stored in a 4°C refrigerator until usage. The broodstock was fed to apparent satiation twice a day until the spawning activity was observed.

The filter cotton in the filtration system was replaced every 3–5 days. Residuals on the bottom of spawning tanks were siphoned once a day. Water was exchanged every 5 days through micro-running water. The water temperature for all tanks was checked twice a day and adjusted as needed.

### 2.5 Sampling and histological and energy analysis

#### 2.5.1 Sampling

Five to eight individuals (at least three females) in each group were randomly collected at four critical stages referring to our previous work (Hussein et al., 2020) (Figure 3), i.e., before the initiation of ovarian redevelopment (Stage 1, before temperature decrease, 16 August), supposed maturation of ovary (Stage 2, right after temperature increase, 25 September), supposed spawning period (Stage 3, 5 weeks after the maintenance of spawning temperature, 31 October), and supposed spawning peak (Stage 4, spawning was observed in all treatment groups except NTNP, 21 November). Body weight, body length, gonad weight, liver weight, mesenteric fat weight, and eviscerated weight were measured following anesthetization using 7–10 mg L^-1^ eugenol. Gonad tissues at four stages were preserved in Bouin’s solution for 48 h and removed into 70% ethanol for histological analysis. The gonadosomatic index (GSI), hepatosomatic index (HSI), and mesenteric fat index (MFI) were expressed as a percent of gonad weight, liver weight, and mesenteric fat weight relative to body weight, respectively. Condition factor (CF) was calculated using the formula CF = 1,000 × (W/L³), where W is body weight (g), and L is body length (cm). Muscle, liver, and gonad tissues at Stage 2 and Stage 4 were collected and preserved at −80°C for energy analysis. Energy changes from gonadal maturation to final maturation can reveal the energy allocation strategies of each treated group and explore the energetic factors affecting the reproductive performance.

#### 2.5.2 Histological analysis

Gonad tissues were dehydrated (80%, 90%, 95%, and double 100% ethanol), cleared (triplicate xylene), immersed and embedded in paraffin, cut at 6–8 μm, stained with hematoxylin and eosin (HE stain), and mounted using neutral resins following routine histological procedures (Shen et al., 2016; 2018; Hussein et al., 2020), with extended processing time (including dehydration, clearing, and paraffin immersion) for ovary samples. Tissue slices were checked and photographed under a light microscope equipped with an imaging system (MShot Image Analysis System, Guangzhou Ming-Mei Technology Co., Ltd.). Long and short axes of oocytes at different stages were measured, and volumes were calculated using the following formula: oocyte volume = πab^2^/6, where a and b are the long and short axes of oocytes, respectively (Shen et al., 2017). At least six glass slides were made, and three slides of ovarian samples for each fish were measured under a light microscope equipped with an imaging system. All oocytes were measured by moving the glass slide back and forth for each sample slide. To establish correlation between the GSI and volume percent of the oocyte at different stages, oocytes were classified into three stages, i.e., primary growth stage, cortical alveoli stage, and vitellogenesis/maturation stage (McMillan, 2007). In addition, ovarian samples that cover different development stages of offspring produced in OSS and in-season spawning (ISS) were also collected and analyzed.

#### 2.5.3 Energy analysis

The energy density (ED) of the muscle, liver, and gonad was determined using the bomb calorimetry method. The wet weight of all samples was recorded and dried in the oven at 85°C until reaching constant weight. Dried samples were ground and crushed using a grinder (Retsch MM400). Caloric content was determined using a Parr6100 oxygen bomb calorimeter (Calorimeter Parr6100, ParrInstrument Company, Moline, IL, United States) following manufacturer instructions. The caloric content of mesenteric fat refers to the energy density of bluegill sunfish (Beuchel et al., 2013), which is a close relative of LMB. Relative total energy (RTE) was calculated by the sum of four main parts of energy storage relative to body weight, including muscle ED multiplied by its dry eviscerated weight, liver ED multiplied by its dry weight, gonad ED multiplied by its dry weight, and mesenteric fat ED multiplied by its dry weight. Four biological repetitions and two technical repetitions were analyzed for all treatment groups of sampling points at Stage 2 and Stage 4.

### 2.6 Data analysis

Data were analyzed using the SPSS program (IBM SPSS Statistics, version 22). The correlation was estimated using a two-tailed Pearson correlation. Best regression was obtained by comparing models generated using curve estimation. Datasets were checked for variance homogeneity and normality before further analysis. The difference in GSI, HSI, MFI, CF, fecundity, and fertilization rate was analyzed by one-way analysis of variance (one-way ANOVA) followed by least significant difference (LSD) *post-hoc* multiple comparisons. Energy density and relative total energy were analyzed by *t*-test. Differences were considered statistically significant when *p* < 0.05. Some of the figures were generated using Origin Pro (version 2018).

## 3 Results

### 3.1 Somatic index variations and energy allocation

**FIGURE 4:**
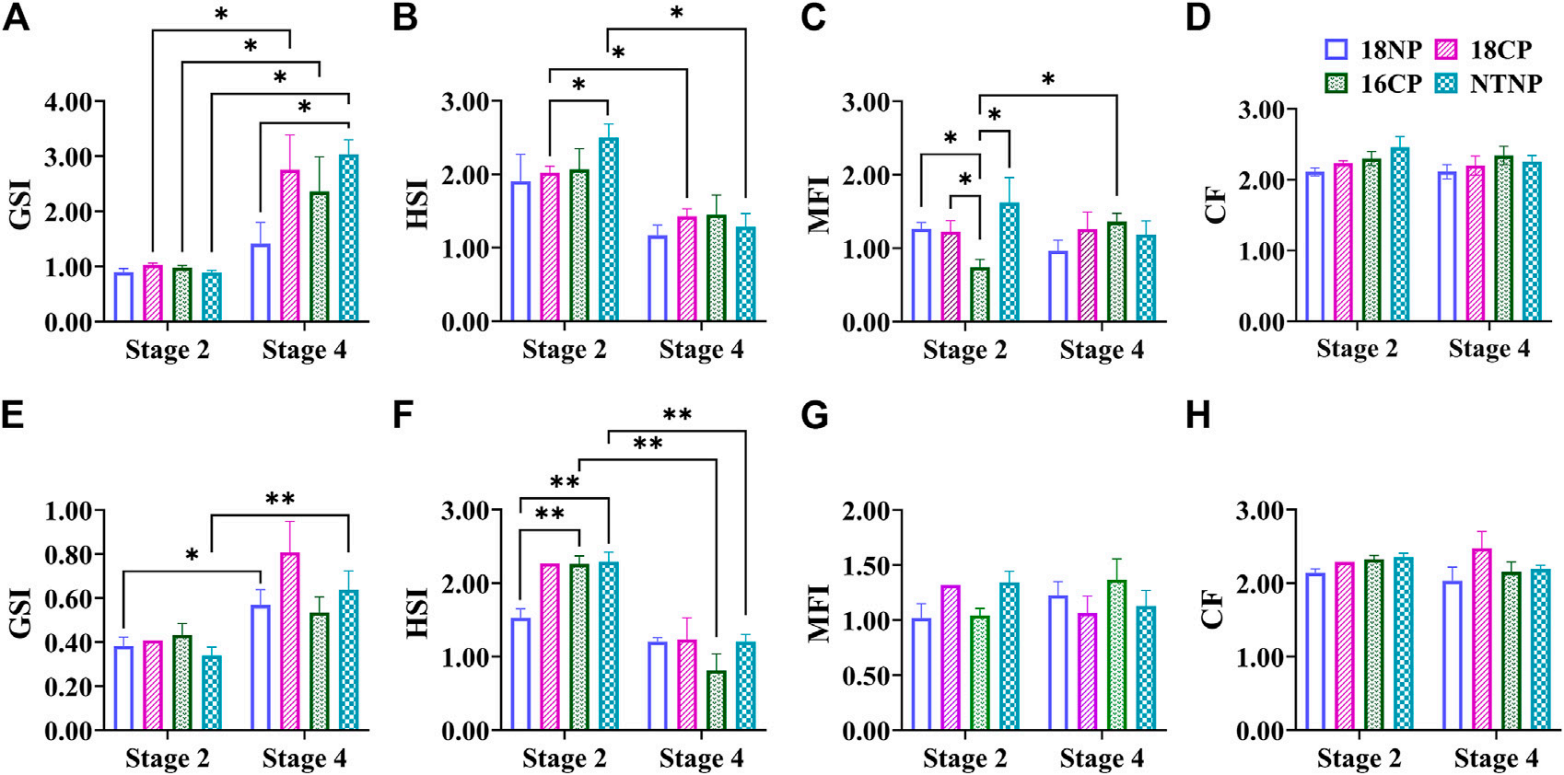
Variations of somatic indexes including GSI, MFI, HSI, and CF during thermo-photoperiod manipulation of largemouth bass. (**A–D)** Female GSI (gonadosomatic index), MFI (mesenteric fat index), HSI (hepatosomatic index), and CF (condition factor). (**E–H)** Male GSI, MFI, HSI, and CF. **Stage 2**, supposed maturation of ovary, right after temperature increase, 25 September. **Stage 4**, the supposed spawning peak, spawning was observed in all treatment groups, on 21 November. These stages were pointed out in Figure 3. Asterisk (*) indicates a significant difference between Stage 2 and Stage 4, or between treatment groups. **18NP**, 18°C overwintering water temperature, natural photoperiod group. **18CP**, 18°C overwintering water temperature, controlled photoperiod group. **16CP**, 16°C overwintering water temperature, controlled photoperiod group. **NTNP**, natural water temperature and natural photoperiod group.

The female GSI of LMB at Stage 4 was significantly higher than that of Stage 2 in all experimental groups except the 18NP group (*p* < 0.05, Figure 4). Interestingly, the female GSI in the NTNP group was significantly higher than that of the 18NP group at Stage 4. The male GSI in 18NP and NTNP groups at Stage 4 was significantly higher than at Stage 2.

In contrast to the GSI, the HSI showed a notable trend of decrease in all experimental groups, indicating the energy consumption of the liver during the maturation and spawning periods. A significant difference in HSI was observed between Stage 2 and Stage 4 in the 18CP and NTNP groups in females, and 16CP and NTNP groups in males, respectively.

The MFI in females and males did not show a clear pattern of variation as observed in the GSI and HSI. For CF, no significant difference was observed between any two groups or between Stage 2 and Stage 4 in either females or males (Figure 4).

**FIGURE 5:**
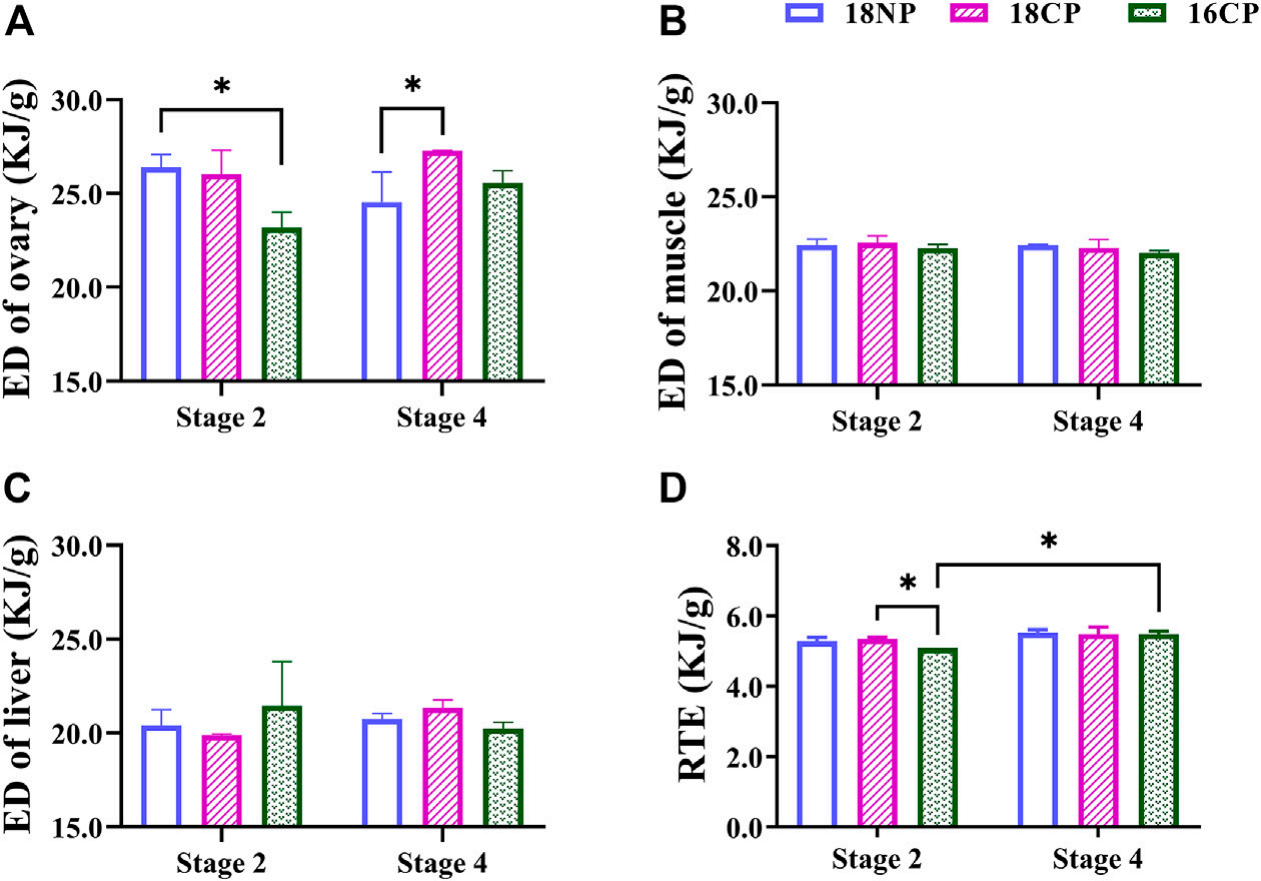
Energy density (ED) of the ovary, muscle, and liver and relative total energy (RTE) of female largemouth bass. **(A)** ED of the ovary. **(B)** ED of muscle. **(C)** ED of the liver. **(D)** RTE of the total individual. RTE was calculated by the sum of four main parts of energy storage relative to body weight, including muscle ED multiplied by dry eviscerated weight, liver ED multiplied by its dry weight, gonad ED multiplied by its dry weight, and mesenteric fat ED multiplied by its dry weight. Asterisk (*) indicates a significant difference between Stage 2 and Stage 4, or between treatment groups. **Stage 2**, the supposed maturation of the ovary, right after the temperature increase, on 25 September. **Stage 4**, the supposed spawning peak, spawning was observed in all treatment groups, on 21 November. **18NP**, 18°C overwintering water temperature, natural photoperiod group. **18CP**, 18°C overwintering water temperature, controlled photoperiod group. **16CP**, 16°C overwintering water temperature, controlled photoperiod group.

To further understand the variations of energy density (ED) in main energy storage organs, we measured the energy content of the ovary, muscle, and liver (Figure 5). The ED of the ovary in the 18NP group was significantly higher than in the 16CP group at Stage 2, and the 18CP group was significantly higher than in the 18NP group, while no significant difference was found between the two stages. Unexpectedly, no significant difference in the ED of the muscle and liver was observed either among groups or between two stages. Relative total energy (RTE) at Stage 4 was significantly higher than Stage 2 in the 16CP group. RTE in the 18CP group was significantly higher than in the 16CP group at Stage 2 (Figure 5).

### 3.2 Gonad development during thermo-photoperiod manipulation

**FIGURE 6:**
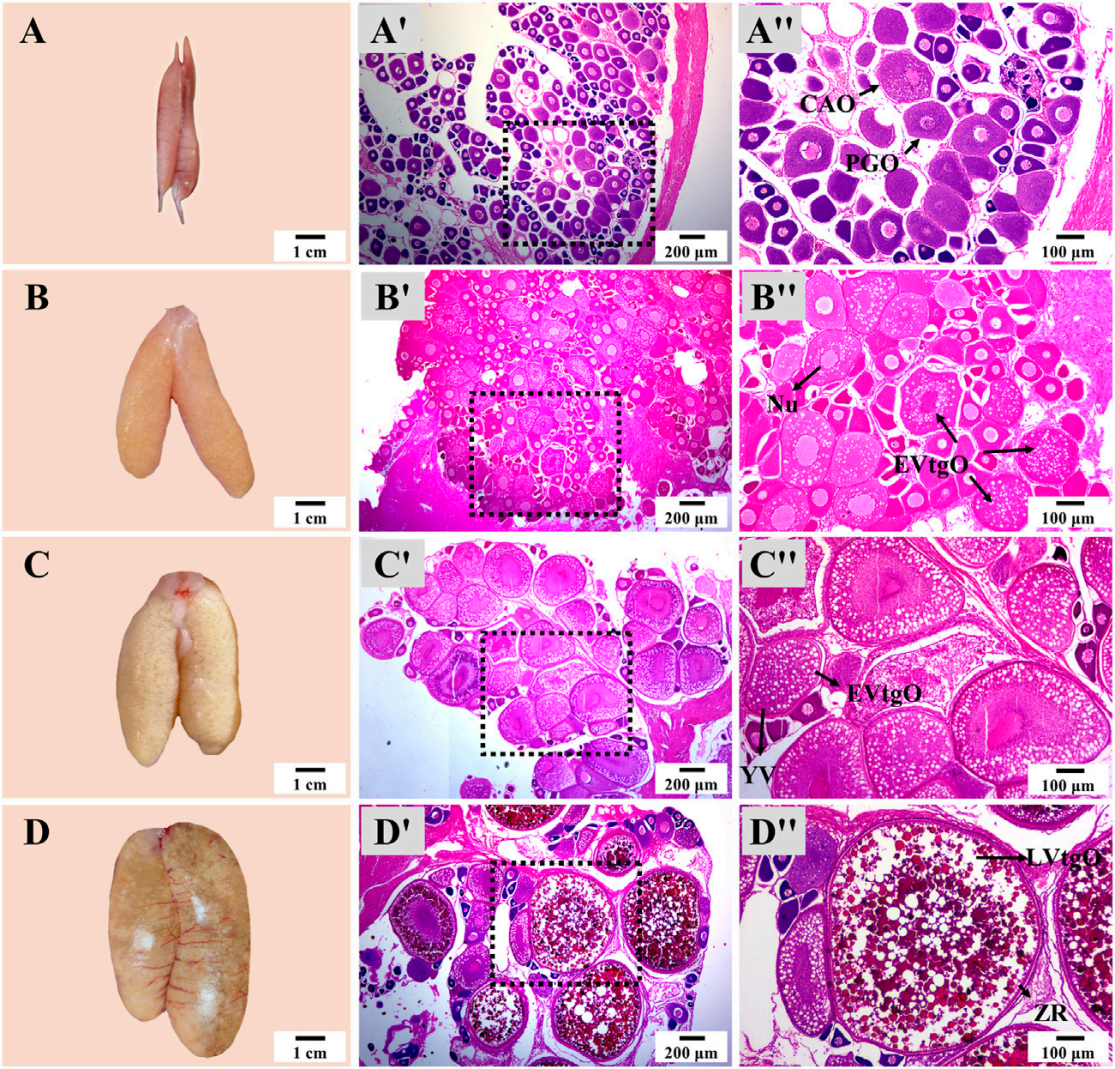
Ovary appearance and histology during thermo-photoperiod manipulation of largemouth bass. **(A–D)** Ovary appearance at Stage 1, Stage 2, Stage 3, and Stage 4, respectively, showing remarkable changes in ovarian size and appearance. **(Aʹ–Dʹ)** Corresponding ovarian histology at Stage 1, Stage 2, Stage 3, and Stage 4, respectively, showing the dramatic changes in the size and composition of oocytes. **(Aʺ–Dʺ)** Represent the enlargement of the dotted box in the histological image of **(Aʹ–Dʹ)**, respectively. **Stage 1**, before the initiation of ovarian redevelopment, before temperature decrease, 16 August. **Stage 2**, supposed maturation of ovary, right after temperature increase, 25 September. **Stage 3**, the supposed spawning period, 5 weeks after the maintenance of spawning temperature, 31 October. **Stage 4**, the supposed spawning peak, spawning was observed in all treatment groups, 21 November. PGO, primary growth stage oocytes. CAO, cortical alveoli stage oocytes. CA, cortical alveoli. EVtgO, early vitellogenesis stage oocytes. LVtgO, late vitellogenesis stage oocytes. YV, yolk vesicles.

There was no notable difference in the appearance and histology of the ovary or testis at the same stage in different groups. The appearance and size of the ovary showed dramatic changes from Stage 1 to Stage 4 during thermo-photoperiod manipulation (Figure 6). At Stage 1, right before the temperature decrease, the ovary appeared to be brownish gray and was mainly composed of primary growth stage oocytes (PGOs) and a few cortical alveoli stage oocytes (CAOs). At Stage 2, right after the temperature increase to spawning temperature (∼23.0°C), the ovary was tan, and different sizes of eggs could be observed with the naked eye. At this time, the ovary was occupied by early vitellogenesis stage oocytes (EVtgOs), CAOs, and PGOs. At Stage 3, 5 weeks after the maintenance of spawning temperature, which is supposed to be the spawning period, the ovary was mainly composed of EVtgOs and a few PGOs. The long axis of the largest oocytes was over 600 μm. At this stage, no spawning was observed in any experimental group. At Stage 4, when spawning was observed in all treatment groups except NTNP, eggs that reached maturation could be observed with the naked eye. The ovary was mainly occupied by mature oocytes, EVtgOs, and a few PGOs. The long axis of the largest oocytes was over 1,300 μm (Figure 6).

**FIGURE 7:**
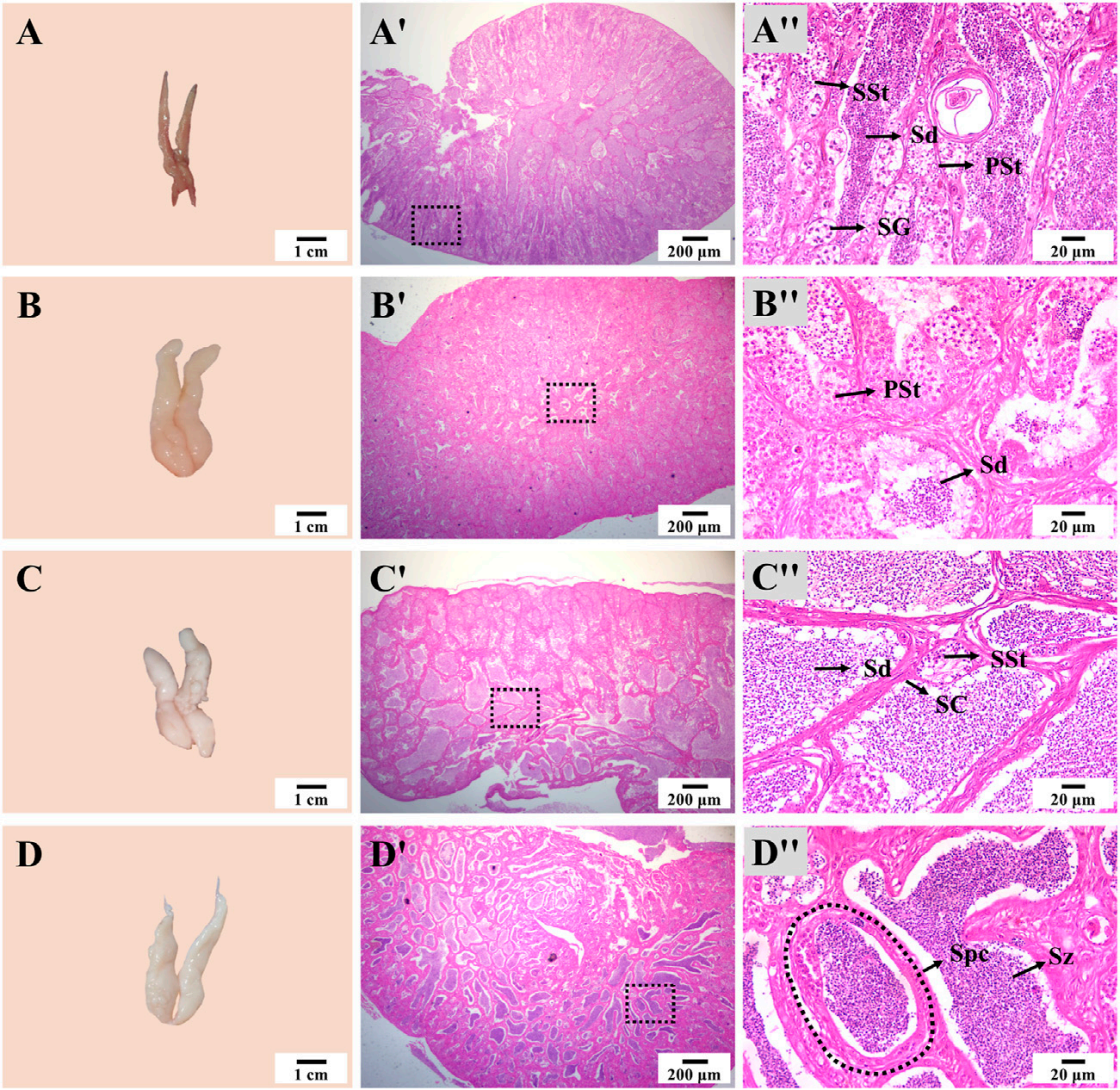
Testis appearance and histology during thermo-photoperiod manipulation of largemouth bass. **(A–D)** Testis appearance at Stage 1, Stage 2, Stage 3, and Stage 4, respectively, showing remarkable changes in testis morphology. **(Aʹ–Dʹ)** Corresponding testis histology at Stage 1, Stage 2, Stage 3, and Stage 4, respectively, showing the dramatic changes in size and composition of spermatocytes. **(Aʺ-Dʺ)** Enlargement of the dotted box in the histological image of **(Aʹ–Dʹ)**, respectively. **Stage 1**, before the initiation of ovarian redevelopment, before temperature decrease, 16 August. **Stage 2**, supposed maturation of ovary, right after temperature increase, 25 September. **Stage 3**, supposed spawning period, 5 weeks after the maintenance of spawning temperature, 31 October. **Stage 4**, spawning was observed in all groups except the NTNP group, 21 November. SG, spermatogonia. PSt, primary spermatocyte. SSt, secondary spermatocyte. Sd, spermatid. Sz, spermatozoa. Spc, spermatocyst. SC, Sertoli cells.

The appearance and size of the testis also showed notable changes during thermo-photoperiod manipulation (Figure 7). At Stage 1, the testis was dark brown and occupied by spermatogonia (SG), primary spermatocyte (PSt), secondary spermatocyte (SSt), and a large number of spermatid (Sd). At Stage 2, right after the temperature increase to spawning temperature, the testis increased in size, and the spermatocyst (Spc) was mainly composed of SSt and Sd. At Stage 3, the off-white testis was occupied by a large number of Sd. At Stage 4, the milk-white testis was full of spermatozoa (Sz), and seminal fluid could be squeezed out by pressing the abdomen (Figure 7).

**FIGURE 8:**
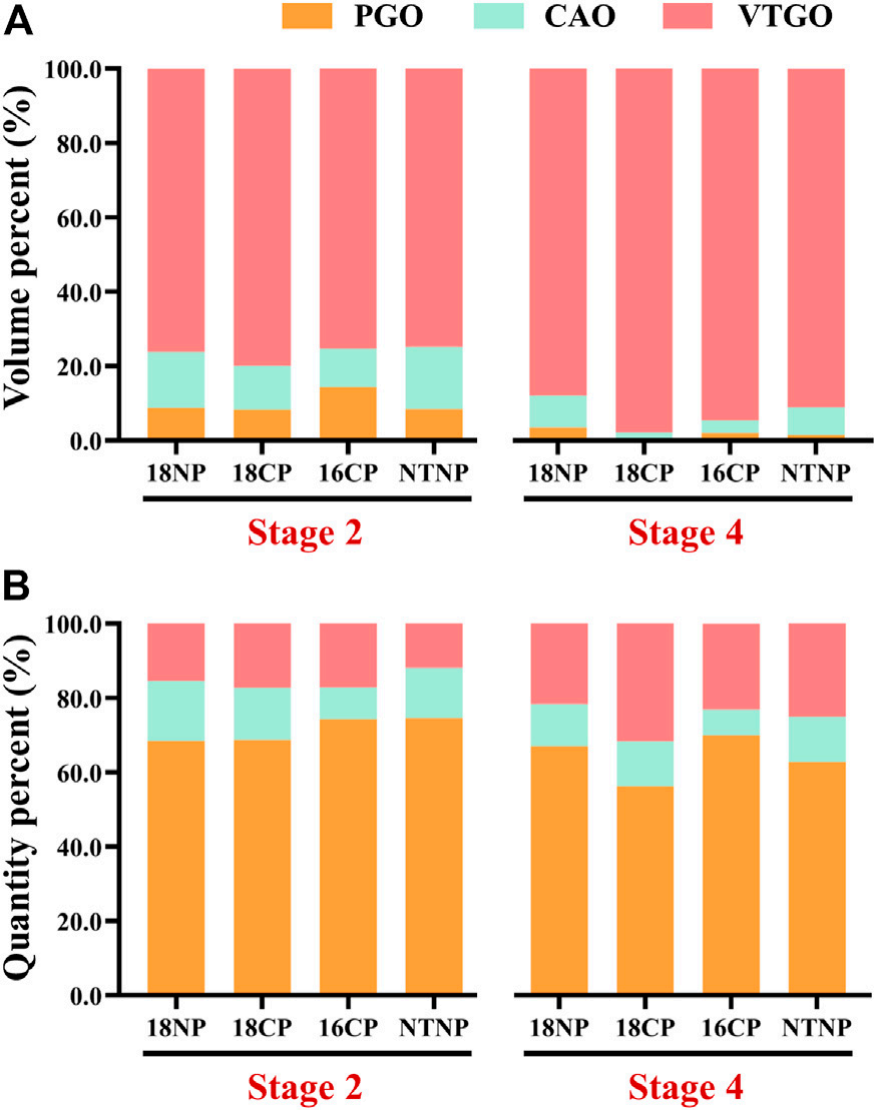
Volume and quantity percentage of primary growth stage oocytes (PGO), cortical alveoli stage oocytes (CAO), and vitellogenesis stage oocytes (VTGO) of largemouth bass. **(A)** Volume percentage of PGO, CAO, and VTGO relative to the total volume of all oocytes. **(B)** Quantity percentage of PGO, CAO, and VTGO relative to the total number of all oocytes. **Stage 2**, supposed maturation of ovary, right after temperature increase, 25 September . **Stage 4**, the supposed spawning peak, spawning was observed in all treatment groups, 21 November. **18NP**, 18°C overwintering water temperature, natural photoperiod group. **18CP**, 18°C overwintering water temperature, controlled photoperiod group. **16CP**, 16°C overwintering water temperature, controlled photoperiod group. **NTNP**, natural water temperature and natural photoperiod group. *N* = 33.

**FIGURE 9:**
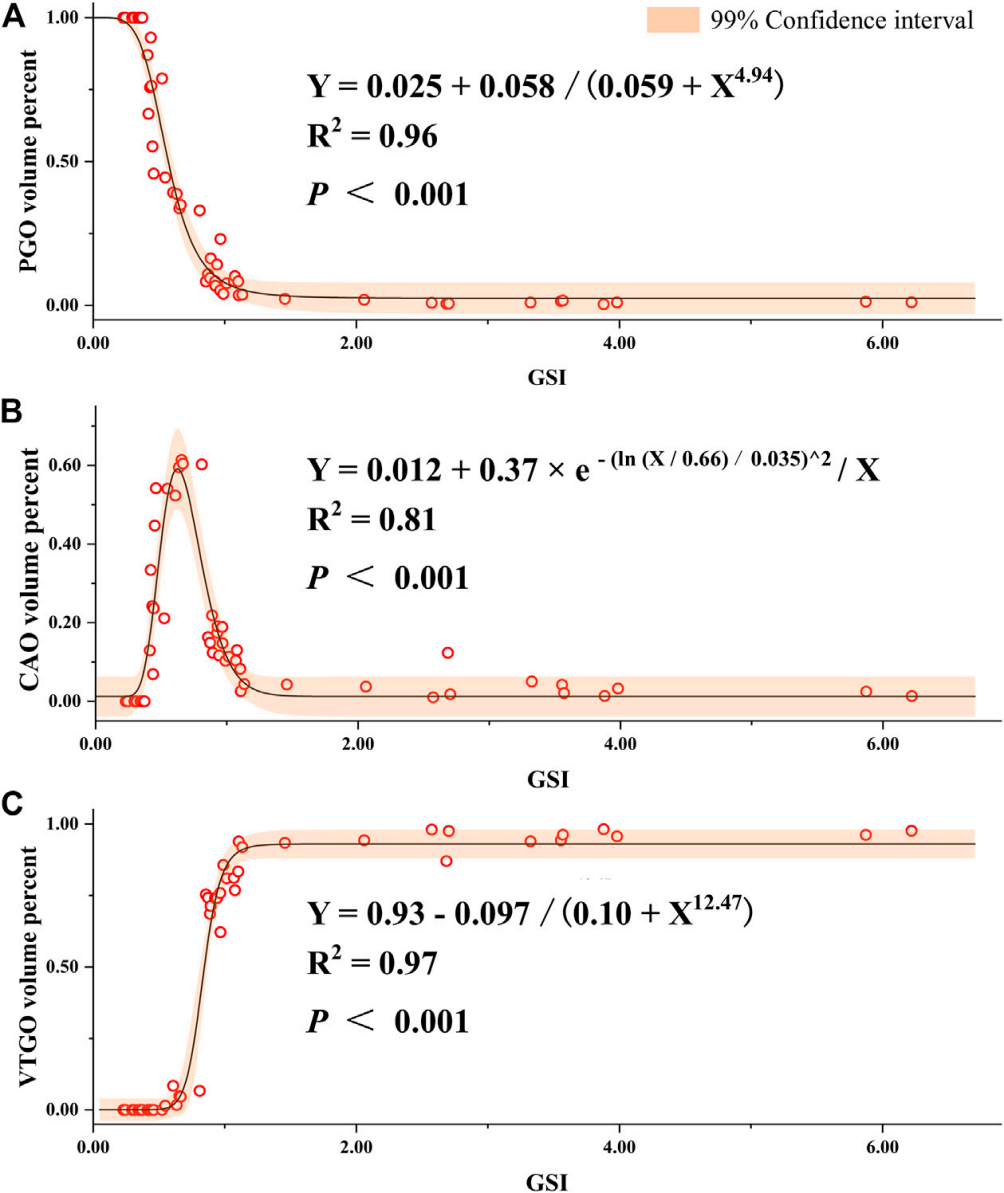
Regression of gonadosomatic index (GSI) and volume percent of different development stages of oocytes in largemouth bass. **(A)** Regression between the GSI and volume percent of PGO (primary growth stage oocytes) relative to the total volume of all oocytes. **(B)** Regression between the GSI and volume percent of CAO (cortical alveoli stage oocytes). **(C)** Regression between the GSI and volume percent of VTGO (vitellogenesis stage oocytes). Oocytes were regarded as an oblate ellipsoid, and the volumes were calculated using the following formula: oocyte volume = πab^2^/6, where a and b are the long and short axes of oocytes, respectively (Shen et al., 2017; Hussein et al., 2020). To represent the volume percent of different stages of oocytes, at least six glass slides were made, and three slides of ovarian samples for each fish were measured. The number of female individuals for the regression is 55, which covers different development stages and ages (1–24 months).

To understand the relationship between the volume/quantity percentage of oocytes and ovary development, we measured the volume and counted the number of all oocytes on slides. Overall speaking, the volume and quantity percentage of VTGO had both increased from Stage 2 to Stage 4 in all groups, and the trend for PGO was the opposite (Figure 8). We further established the regression between volume percentages of PGO/CAO/VTGO and GSI using data from different development stages of ovaries (Figure 9). Interestingly, PGO/CAO/VTGO volume percentage showed a good correlation with the GSI. For example, the regression equation of both PGO and VTGO volume percentage and GSI showed an inversely proportional function with excellent *R* ^2^ values (0.96 and 0.97, respectively). Meanwhile, as a transitional state of oocytes, CAO volume increased first and dramatically decreased as ovary development. It is worth mentioning that PGO and VTGO volume percentages remained stable when the GSI was close to 2 (Figure 9), probably indicating the maturation of the ovary.

### 3.3 Comparative analysis of OSS and ISS

OSS was first observed on 17November in the 16CP group, 6 weeks after the water temperature increased to the spawning temperature (23.0°C). The last spawning was found in the 18CP group on 2 January when the temperature decreased to 17.0°C (Figure 3). Nine spawned nests were observed in total during the 3 months when the water temperature was maintained at spawning temperature for OSS.

ISS was first observed on 28th March, 1 week after the water temperature was increased to the spawning temperature (23.0°C), which is a half month earlier than the natural spawning in outside ponds (Cui et al., 2021). Fifty six spawned nests were observed in total during the 1 month when the water temperature was maintained at the spawning temperature for ISS. The spawning activity was artificially ceased by decreasing water temperature because there were too many spawned nests, which was beyond our processing capacity.

**FIGURE 10:**
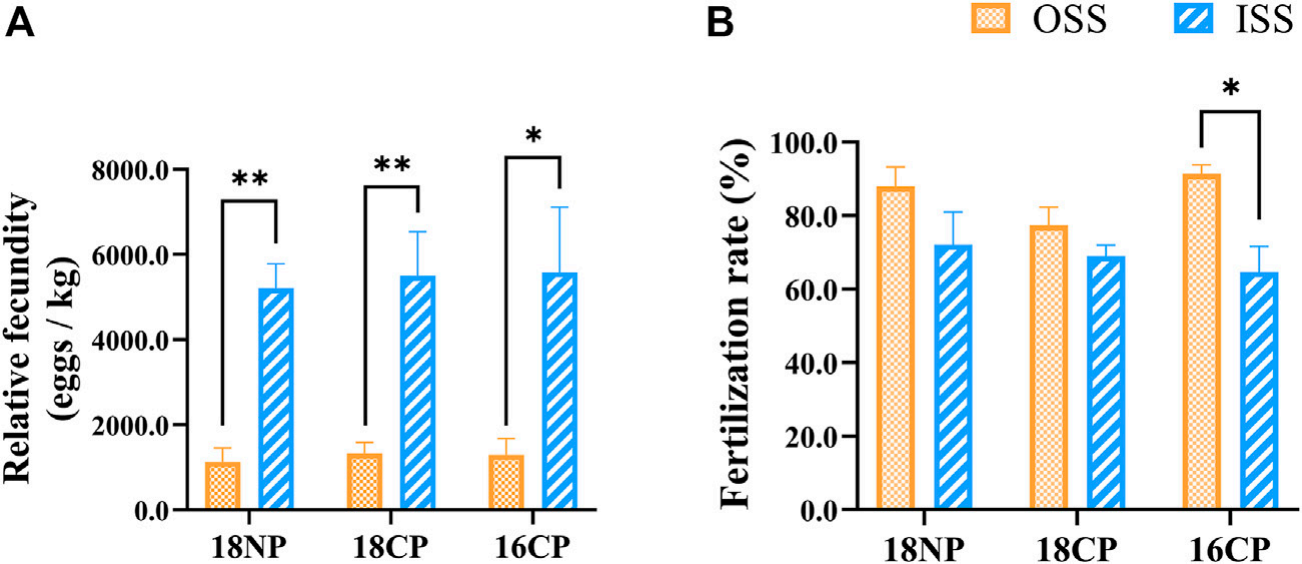
Relative fecundity and fertilization rate in out-of-season spawning (OSS) and in-season spawning (ISS). **(A)** Relative fecundity, i.e., spawned egg numbers relative to female weight. **(B)** Fertilization rate. **18NP**, 18°C overwintering water temperature, natural photoperiod group. **18CP**, 18°C overwintering water temperature, controlled photoperiod group. **16CP**, 16°C overwintering water temperature, controlled photoperiod group. The broodstock of ISS came from the same group of fish in OSS. Asterisk (*) and double asterisk (**) indicate significant (*p* < 0.05) and extremely significant (*p* < 0.01) differences between OSS and ISS in each group, respectively.

Relative fecundities (spawned eggs per kilogram female) of OSS were significantly lower than those of ISS in all three groups (18NP, 18CP, and 16CP). Interestingly, the fertilization rates of OSS (85.6%) were significantly higher than those of ISS (68.6%) when the data of the three groups were combined (*p* < 0.01). In the 16CP group, the fertilization rate of OSS is significantly higher than that of ISS, while no significant difference was observed in the other two groups (Figure 10). Finally, 25,000 and 66,000 fry were produced in OSS (45 females) and ISS (30 females), respectively, in the small recirculating system.

### 3.4 Spawning nest preference

**FIGURE 11:**
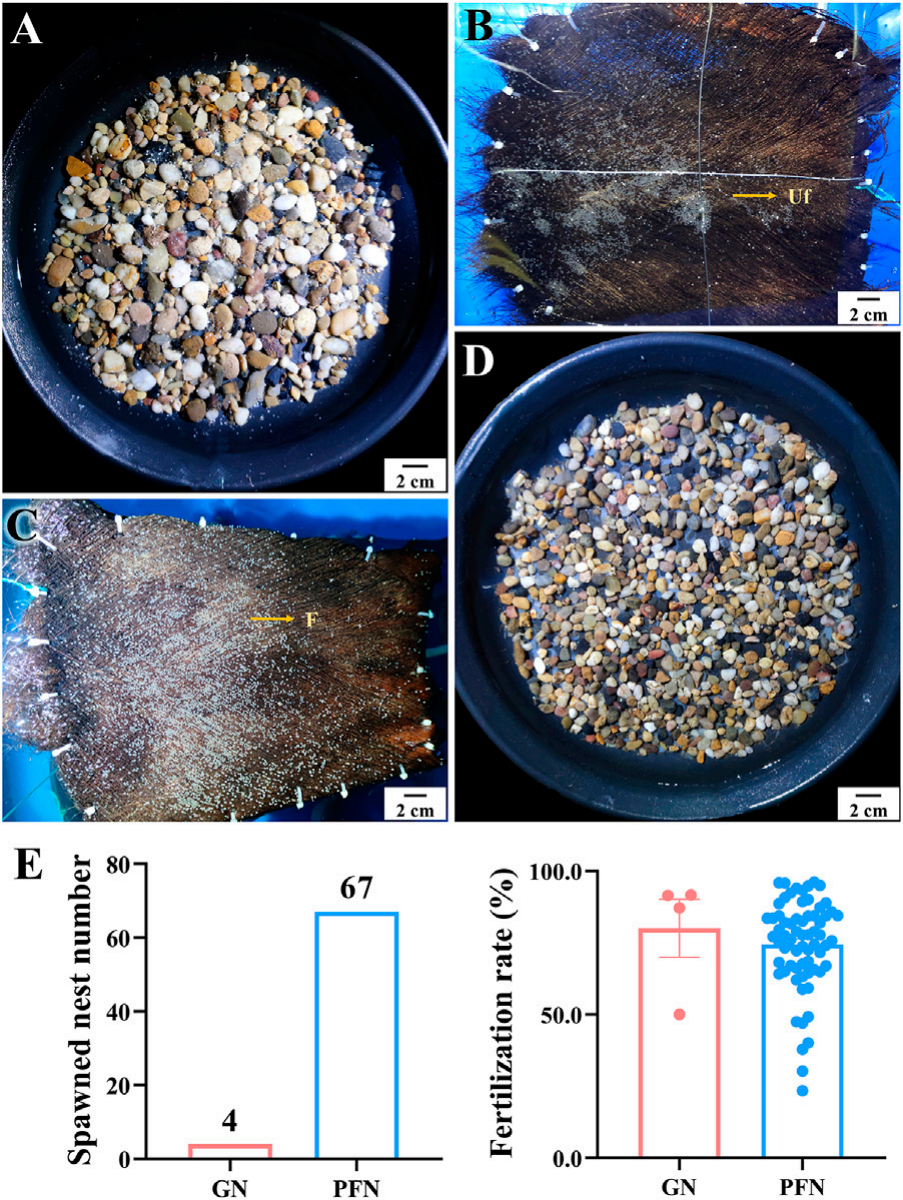
Spawning nest preference and fertilization rates on palm fiber nest and gravel nest of largemouth bass. **(A, B)** Spawned eggs on gravel nest (GN) and palm fiber nest (PFN) in out-of-season spawning, respectively. **(C, D)** Spawned eggs on PFN and GN in in-season spawning, respectively. **(E)** Comparison of spawned nest numbers and fertilization rate between GN and PFN.

Two palm fiber nests (PFNs) and two gravel nests (GNs) crisscrossed in each spawning tank to distinguish the spawning nest preference of LMB. Sixty-seven and four spawned PFN and GN were observed, respectively, indicating a notable preference of LMBs for PFNs (Figure 11). There was no significant difference in the fertilization rate of spawned eggs between PFNs and GNs.

## 4 Discussion

### 4.1 OSS succeeded in a low-cost and controllable recirculating system

In the present work, we designed a low-cost and controllable recirculating system and successfully realized OSS through a short period of thermo-photoperiod manipulation in LMB. Optimization of thermo-photoperiod control and transformation of large-scale OSS and out-of-season production will be greatly helpful in the high-quality development of the LMB industry, by changing the culture cycle, relieving centralized production and selling, and finally increasing profit.

In the current work, the small and low-cost controllable recirculating system allowed stable and accurate control of temperature and photoperiod according to the records (Figure 3). The rearing density during the chilling period of water temperature was higher than during the warming and spawning periods, which was also a good way of saving electric power. Meanwhile, the system also allows ovary development, maturation, and OSS in all thermo-photoperiod-controlled groups. Finally, we produced 25,000 fry using 45 females in the OSS, where efficiency was higher than in our previous work that produced 480,000 fry using nearly 4,000 females (Hussein et al., 2020). We cultured OSS-produced fry and successfully weaned them to formula feed during 12–20 days post-hatching. The survival rate was about 60% on average, and the deformity rate was less than 5% until 60 days post-hatching, indicating the good quality of the OSS-produced fry.

**FIGURE 12:**
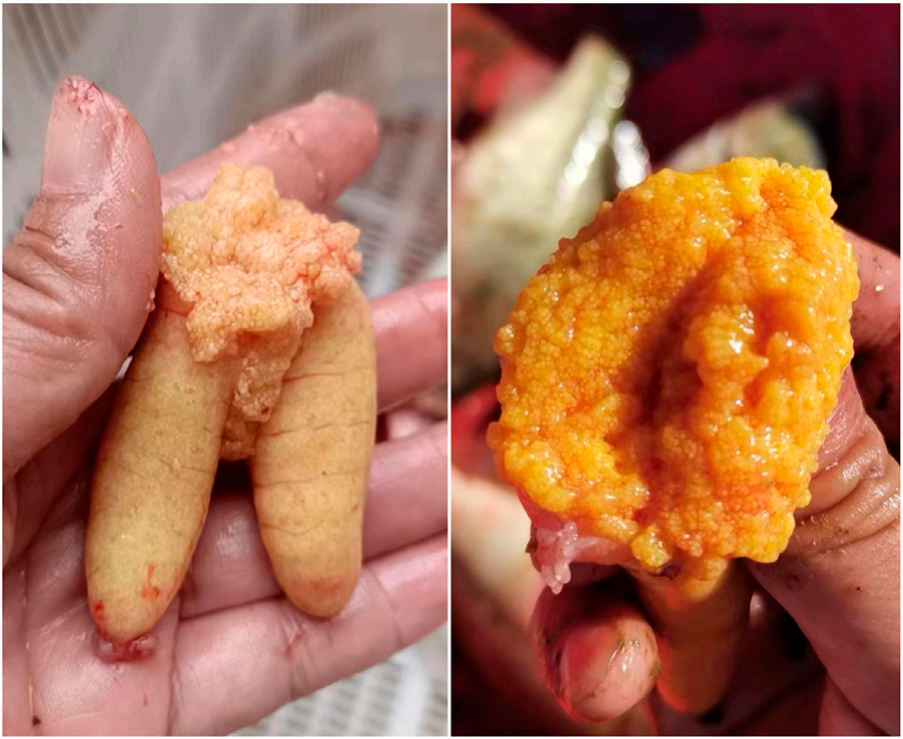
Ovary reached maturation on 8 October, 2022, in the largemouth bass individuals that were produced during October and December of the last year. The out-of-season spawning produced largemouth bass that underwent 20°C–28°C water temperature and natural photoperiod during their lifetime before reaching maturation. Photos were taken by Ze-Xiao Zhou, an out-of-season spawning-produced largemouth bass farmer.

Intriguingly, some LMB farmers have found that the ovary developed very well and reached maturation during October and December in most individuals produced during October and December of the last year (Figure 12), applying a similar regime of OSS of the present work. In addition, some LMB breeding farms transferred these mature fish to spawning ponds, and they had been spawning 1 week later. Therefore, the results of the current work provide an important foundation for continuous low-cost OSS in LMB.

### 4.2 Energy expenditure of stored fat may be the key for large-scale OSS

OSS has been achieved in several fish species, and environmental control (mainly by temperature and/or photoperiod manipulation) of fish reproduction has been reviewed by many researchers (Bromage et al., 2001; Lubzens et al., 2009; Migaud et al., 2010; Taranger et al., 2010; Wang et al., 2010; Migaud et al., 2013). The direction of thermo-photoperiod changes, the amplitude, rates, and timing of variations of each period (e.g., chilling, low-temperature holding, and warming) are considered to influence the quality of spawning (e.g., fecundity, fertilization rate, and offspring quality). However, energy allocation, which has been regarded as the threshold for successful reproduction (Migaud et al., 2010), has been paid little attention in the research of OSS.

In our previous work about the annual ovary development of LMB in outdoor earthen ponds, we found a good negative correlation between the GSI and HSI/MFI starting from the initiation of ovary development until spawning (September to April of the next year) (Cui et al., 2021). During the period of initiation, vitellogenesis, and the maturation of oocyte development, HSI and MFI had been decreasing from about 3.0 to 2.1 and then reached the minimum (1.5 and 0.6) at the beginning of April, right before the GSI reached the maximum (∼8.0) and first spawning was observed (Cui et al., 2021). The MFI was also found to decrease to the minimum before the spawning events of the natural LMB populations (Brown and Murphy, 2004). In the present work, however, even though HSI decreased at Stage 4 to the level (1.4) that was similar to natural spawning, MFI did not show apparent changes (Figure 4). In addition, the energy density of the liver did not show notable changes from Stage 2 to Stage 4 (Figure 5). These results indicate that the mesenteric fat has not been sufficiently consumed and transformed for ovary development and maturation.

Substantial evidence has proved that moderate food restriction and low MFI are important for the reproduction of farmed fish (El-Sayed and Kawanna, 2008; Higuchi et al., 2018; Cardona et al., 2019; Hu et al., 2021; Xiong et al., 2022). For example, the fecundity, fertilization rate, and fry production per female of two low-MFI groups (0.56 and 1.97) were significantly higher than those of two high-MFI groups (5.92 and 9.62) in yellow catfish (Hu et al., 2021). In addition, females with high MFI were difficult to be artificially spawned, and the moderate feeding rate group showed high fecundity, low mortality, and high fertilization and hatching rate in this species (Xiong et al., 2022). Therefore, proper starvation during the period of thermo-photoperiod manipulation is important for improving the fecundity of OSS. Importantly, a substantial decrease in MFI should be treated as the most important signal for the initiation of OSS.

### 4.3 Volume percentage of oocytes is a better indicator of gonadal development and maturation

The GSI has been used as a widespread metric of gonadal development and maturation. However, it is not always a precise index because of its high amplitude, particularly when a given fish underwent diverse environmental conditions (e.g., food quality and quantity, space, competition, and annual thermo-photoperiod changes) (Devlaming et al., 1982; Packard and Boardman, 1988; Shen et al., 2017).

In the present work, we found a close relationship between the volume percentage of different stages of oocytes and gonad development, according to further analysis of a large number of histological images covering different ovarian development stages. We, then, established a significant regression between the PGO/CAO/VTGO volume percentage and GSI (Figure 9). These results, combined with the findings of GSI variations (Figure 4), suggest that the volume percentage of oocytes is a better indicator of gonadal development and maturation. For example, we have found that the GSI was 1.5–3.5 when the spawning activity was observed. At the same time, we also found that the VTGO volume percentage reached the maximum when the GSI just reached 2.0. In other words, VTGO volume percentage can indicate when the ovary reaches maturation but the GSI cannot. We have noticed that the female GSI of mature LMBs varied dramatically in different conditions, from <2 to 14 (Rosenblum et al., 1999; Martyniuk et al., 2009; 2013; Hussein et al., 2020). Therefore, a more precise index of ovarian development and maturation, i.e., PGO/CAO/VTGO volume percentage, will be greatly helpful for the successful reproduction in LMBs and other fish.

### 4.4 LMB prefers palm fiber as the spawning nest

It was reported that LMBs spawned on sandstones, gravels, rock ledges, or silt in natural water bodies (Miller, 1971; Nack et al., 1993; Fujimoto et al., 2021). In the breeding industry of LMB, gravel nests and palm fiber nests are the most frequently used bottom types. In the present work, however, we found that LMB showed an evident preference for palm fiber as the spawning nest compared to gravel (Figure 11). In fact, a PFN is more convenient and practical for egg collection, hatching, disinfection, and reuse. Recently, industrialized breeding, hatching, nursing, and culture of LMBs are greatly needed because of the strong price fluctuation (Hussein et al., 2020), epidemic viral disease (Zhu et al., 2020; Zeng et al., 2022), and unstable culture environmental conditions of earthen ponds. The determination of spawning nest preference provides important information for the convenient setting, collection, hatching, and disinfection of spawning nests in indoor LMB breeding.

## 5 Conclusion and further research

In conclusion, we designed a controllable and low-cost recirculating system for LMB. The system allowed OSS at the end of a year and advanced re-spawning in the next spring. The investigation of somatic indexes, gonad development, and spawning strongly suggests that adequate energy expenditure of stored fat may determine the success and fecundity of OSS. We also found that the profiling of the volume percentage of oocytes is a better index of gonad development and maturation in LMB. The findings of this work provide important technique guidance for practical OSS of the LMB aquaculture industry and standardization of ovary development and maturation in fish with asynchronous developmental oocytes.

Further research will focus on the effects of starvation, space, and efficient thermo-photoperiod on OSS performance. The research on ovary development and maturation of OSS-produced fish will be greatly helpful for continuous OSS with low cost for the LMB industry.

## Data Availability

The original contributions presented in the study are included in the article/Supplementary Materials, further inquiries can be directed to the corresponding author.
